# Supplementary material for: Scaling of inertial delays in terrestrial mammals
Source: PLoS One. 2020 Feb 4;15(2):e0217188. doi: 10.1371/journal.pone.0217188 (PMC6999919; doi:10.1371/journal.pone.0217188)
Supplement: S1 File — In this document, we have provided detailed derivations for our analytical calculations, described the Monte Carlo simulations used to determine confidence intervals, and elaborated on the methods for determining sensitivity to muscle torque. It contains the following sections: A. Analytical derivation of fall time B. Analytical derivation for the swing task C. Analytical derivation for the posture task D. Determining confidence intervals E. Effect of changing muscle torque on inertial delay scaling. (DOCX) [file pone.0217188.s001.docx]

# ­Supplementary Material

Scaling of inertial delays in terrestrial mammals

Sayed Naseel Mohamed Thangal and J. Maxwell Donelan

PLOS ONE

# A. Analytical derivation of fall time

To analytically derive the scaling of fall time, consider an animal of mass $M$*,* falling from the height of its leg $L$ to the floor. $F_{g}$ is the force of gravity and $g$ is the acceleration due to gravity. The equations for acceleration $\ddot{y}(t)$, velocity $\dot{y}(t)$ and position $y(t)$ are:

$$\begin{aligned} \ddot{y}\left( t \right)=\frac{d^{2}y}{{dt}^{2}}=\frac{F_{g}}{M}=\frac{-gM}{M}=-g\#\left( S1 \right) \end{aligned}$$

$$\begin{aligned} \dot{y}\left( t \right)=\int\ddot{y}dt=-gt+\dot{y}_{0}\#\left( S2 \right) \end{aligned}$$

$$\begin{aligned} y\left( t \right)=\int\dot{y}dt=\frac{-gt^{2}}{2}+\dot{y}_{0}t+y_{0}\#\left( S3 \right) \end{aligned}$$

The initial velocity $\dot{y}_{0}$ is 0 and initial position$y_{0}$is $L$. Assuming geometric similarity, leg length scales with $M^{1/3}$. Then the time $t_{f}$ required for an animal to fall to the floor is:

$$\begin{aligned} t_{f}=\sqrt{\frac{2L}{g}}\propto\sqrt{L}\propto M^{1/6}\#\left( S4 \right) \end{aligned}$$

# B. Analytical derivation for the swing task

The swing task represents an animal repositioning its swing leg to control foot placement and maintain stability during walking and running. For the swing task, the pendulum is required to move from rest at the origin, to a final angle $\theta_{f}$ under the control of muscle torque $T_{musc}$. We ignore gravity to simplify the calculations. $\Delta\theta=(\theta_{f}-0)$, represents the movement magnitude.

The fastest way to complete this movement is to apply a constant torque to accelerate from 0 to $\theta_{f}/2$, then reverse the direction of torque to decelerate and stop at $\theta_{f}$. Since the movement is symmetrical, we consider only the first half from 0 to $\theta_{f}/2$. The equations for the angular acceleration $\ddot{\theta}(t)$, angular velocity $\dot{\theta}(t)$ and angle $\theta(t)$ are:

$$\begin{aligned} \ddot{\theta}\left( t \right)=\frac{d^{2}\theta}{{dt}^{2}}=\frac{T_{musc}}{ML^{2}}\#\left( S5 \right) \end{aligned}$$

$$\begin{aligned} \dot{\theta}\left( t \right)=\int\ddot{\theta}dt=\frac{T_{musc}}{ML^{2}}t+\dot{\theta}_{0}\#\left( S6 \right) \end{aligned}$$

$$\begin{aligned} \theta\left( t \right)=\int\dot{\theta}dt=\frac{T_{musc}}{2ML^{2}}t^{2}+\dot{\theta}_{0}t+0=\frac{\theta_{f}}{2}\#\left( S7 \right) \end{aligned}$$

Because the initial velocity $\dot{\theta}_{0}$­is 0, and our desired final angle is $\theta_{f}/2$, we can rearrange Eqn S7 to solve for $t$. The total inertial delay is twice this time to account for the time spent in each half of the total movement:

$$\begin{aligned} t_{ID}=2\sqrt{\frac{ML^{2} \theta_{f}}{T_{musc}}}\#\left( S8 \right) \end{aligned}$$

The applied muscle torque $T_{musc}$ is the product of a constant muscle moment arm $R_{musc}$ and the muscle force $F_{musc}$.

$$\begin{aligned} T_{musc}=F_{musc}R_{musc}\#\left( S9 \right) \end{aligned}$$

We assume that the muscle moment arm scales with geometric similarity ($M^{1/3}$).

If muscle force scales with dynamic similarity ($F_{musc}\propto M^{1}$), we can determine the scaling of inertial delay by substituting Eqn S9 into Eqn S8:

$$\begin{aligned} t_{ID}=2\sqrt{\frac{ML^{2}\theta_{f}}{F_{musc}R_{musc}}}=2\sqrt{\frac{c_{1}M^{5/3}\theta_{f}}{{c_{2} M}^{4/3}}}\propto M^{1/6}.\sqrt{\Delta\theta}\#\left( S10 \right) \end{aligned}$$

where $c_{1}$ and $c_{2}$ are constants of proportionality.

$$\begin{aligned} t_{rel}=\frac{t_{ID}}{t_{char}}\propto\frac{M^{1/6}}{M^{1/6}}\propto M^{0}\#\left( S11 \right) \end{aligned}$$

where $t_{ID}$ is the inertial delay, $t_{char}$ is the characteristic movement time, and $t_{rel}$ is the relative delay.

Instead, if muscle forces scale with cross sectional area ($F_{musc}\propto M^{2/3}$), getting relatively weaker with increases in size, inertial delay scales as:

$$\begin{aligned} t_{ID}=2\sqrt{\frac{ML^{2}\theta_{f}}{F_{musc}R_{musc}}}=2\sqrt{\frac{{c_{1}M}^{5/3}\theta_{f}}{{c_{2}M}^{1}}}\propto M^{1/3}.\sqrt{\Delta\theta}\#\left( S12 \right) \end{aligned}$$

$$\begin{aligned} t_{rel}=\frac{t_{ID}}{t_{char}}\propto\frac{M^{1/3}}{M^{1/6}}\propto M^{1/6}\#\left( S13 \right) \end{aligned}$$

# C. Analytical derivation for the posture task

The posture task models a standing animal recovering its balance after being perturbed. We represent the standing quadruped with a pendulum, which starts from an initial position and has an initial clockwise velocity in the sagittal plane due to a perturbation pushing it forward. We define inertial delay as the time required for muscle torque to return the pendulum to rest back at the initial position after recovering from the perturbation. We again use the simple model (Fig 1) and ignore the effects of gravity. For this task, the movement is not symmetrical. To analytically derive the equations for inertial delay in the posture task, it is convenient to break down the movement into three phases: A, B and C. In phase A, the pendulum starts at an initial position with a clockwise velocity due to the perturbation. We then apply a counter-clockwise torque to decelerate the pendulum and reject the velocity perturbation, stopping at a clockwise angle. In phase B, we continue to apply the counter-clockwise torque, accelerating the pendulum from rest with a counter-clockwise velocity as it moves back towards its initial position. In phase C, we switch the torque direction again so that a clockwise torque now decelerates the pendulum and brings it to rest at the initial position, thereby completing the response to the velocity perturbation. We describe the analytical derivation below.

In phase A, the pendulum starts from an initial angle${}_{A}{\theta_{0}}$ with an initial clockwise angular velocity $-{}_{A}{\dot{\theta}_{0}}$ . We define${}_{A}{\theta_{0}}$to be the origin with value 0, and counter-clockwise movements to be positive. We then apply a counter-clockwise torque $T_{musc}$ that brings the pendulum to rest at a final position$-{}_{A}{\theta_{f}}$. The equations for angular acceleration ${}_{A}{\ddot{\theta}(t)}$, angular velocity ${}_{A}\dot{\theta}(t)$ and angle ${}_{A}\theta(t)$ are:

$$\begin{aligned} {}_{A}{\ddot{\theta}\left( t \right)}=\frac{d^{2}\theta}{{dt}^{2}}=\frac{T_{musc}}{ML^{2}}\#\left( S14 \right) \end{aligned}$$

$$\begin{aligned} {}_{A}\dot{\theta}\left( t \right)=\int{}_{A}{\ddot{\theta}dt}=\frac{T_{musc}}{ML^{2}}{}_{A}t-{}_{A}{\dot{\theta}_{0}}=0\#\left( S15 \right) \end{aligned}$$

$$\begin{aligned} {}_{A}\theta\left( t \right)=\int{}_{A}\dot{\theta}dt=\frac{T_{musc}}{2ML^{2}}{{}_{A}t}^{2}-{}_{A}{\dot{\theta}_{0}}{}_{A}t+0=-{}_{A}{\theta_{f}}\#\left( S16 \right) \end{aligned}$$

Rearranging Eqn S15, we can express the duration of phase A $({}_{A}t)$ in terms of the initial velocity of the perturbation:

$$\begin{aligned} {}_{A}t=\frac{ML^{2}{}_{A}{\dot{\theta}_{0}}}{T_{musc}}\#\left( S17 \right) \end{aligned}$$

Substituting Eqn S17 into Eqn S16 and simplifying gives us the final angle of phase A (${}_{A}{\theta_{f}}$) in terms of the initial velocity${}_{A}{\dot{\theta}_{0}}$:

$$\begin{aligned} {}_{A}{\theta_{f}}=\frac{ML^{2}{}_{A}{\dot{\theta}_{0}^{2}}}{2T_{musc}}\#\left( S18 \right) \end{aligned}$$

In phases B & C, the pendulum is brought back to rest at the origin from the end position of phase A ($-{}_{A}{\theta_{f}}$). The movements in phases B and C are equal and opposite, with a counter-clockwise torque initially accelerating the pendulum from rest to a position of $-{}_{A}{\theta_{f}}/2$ in phase B, followed by a clockwise torque decelerating the pendulum over the same angular distance to rest at the origin in phase C. Therefore, we only need to evaluate the time required for phase B, since the time required for phase C will be the same. The subtask to be accomplished in phases B and C is the same as that of the entire swing task in section 3.3—begin at rest, move through some angular displacement, and end at rest. The additional feature of the posture task is that the initial velocity perturbation determines the subsequent angular displacement. The equations for the angular acceleration ${}_{B}{\ddot{\theta}(t)}$ , angular velocity ${}_{B}\dot{\theta}(t)$ and angle ${}_{B}\theta(t)$ are:

$$\begin{aligned} {}_{B}{\ddot{\theta}\left( t \right)}=\frac{d^{2}\theta}{{dt}^{2}}=\frac{T_{musc}}{ML^{2}}\#\left( S19 \right) \end{aligned}$$

$$\begin{aligned} {}_{B}\dot{\theta}\left( t \right)=\int{}_{B}\ddot{\theta}dt=\frac{T_{musc}}{ML^{2}}{}_{B}t+0\#\left( S20 \right) \end{aligned}$$

$$\begin{aligned} {}_{B}\theta\left( t \right)=\int{}_{B}\dot{\theta}dt=\frac{T_{musc}}{2ML^{2}}{{}_{B}t}^{2}+0-{}_{A}{\theta_{f}}=\frac{- {}_{A}{\theta_{f}}}{2}\#\left( S21 \right) \end{aligned}$$

Simplifying Eqn S21 gives:

$$\begin{aligned} \frac{{}_{A}{\theta_{f}}}{2}=\frac{T_{musc} {{}_{B}t}^{2}}{2ML^{2}}\#\left( S22 \right) \end{aligned}$$

Substituting the final angle of phase A (${}_{A}{\theta_{f}}$) from Eqn S18 into Eqn S22 and solving for the duration of phase B (${}_{B}t$) gives:

$$\begin{aligned} {}_{B}t=\frac{ML^{2} {}_{A}{\dot{\theta}_{0}}}{{\sqrt{2} T}_{musc}}\#\left( S23 \right) \end{aligned}$$

Solving for the total time for the whole motion (phases A, B and C) gives:

$$\begin{aligned} t_{ID}={}_{A}t+2{}_{B}t=\left( 1+\sqrt{2} \right)\frac{ML^{2} {}_{A}{\dot{\theta}_{0}}}{T_{musc}}\#\left( S24 \right) \end{aligned}$$

Since larger animals have heavier bodies, longer limbs and larger muscles, we scaled the size of the perturbation with animal mass to evoke responses with similar relative magnitude. To do this, we express the initial angular velocity of the pendulum, representing the applied perturbation, in terms of linear velocity:

$$\begin{aligned} {}_{A}{\dot{\theta}_{0}}=\frac{v}{L}\#\left( S25 \right) \end{aligned}$$

where $v$ is the linear velocity caused by the initial perturbation and $L$ is the length of the pendulum. We perturbed each model using an initial linear velocity scaled based on a constant dimensionless velocity $v_{ND}$ [1]:

$$\begin{aligned} v_{ND}=\frac{v}{\sqrt{gL}} , v=v_{ND}\sqrt{gL}\propto M^{1/6}\#\left( S26 \right) \end{aligned}$$

Substituting the values for ${}_{A}{\dot{\theta}_{0}}$ from Eqn S25 and $T_{musc}$ from Eqn S9 into Eqn S24 and assuming muscle force scales with dynamic similarity ($F_{musc}\propto M^{1}$) predicts that the total time for the posture task scales as:

$$\begin{aligned} t_{ID}={}_{A}t+2{}_{B}t=\left( 1+\sqrt{2} \right)\frac{ML^{2}v}{F_{musc}R_{musc}L}=\frac{c_{1}M^{1}M^{2/3}M^{1/6}}{c_{2}M^{1}M^{1/3}M^{1/3}}\propto M^{1/6}.v_{ND}\#\left( S27 \right) \end{aligned}$$

$$\begin{aligned} t_{rel}=\frac{t_{ID}}{t_{char}}\propto\frac{M^{1/6}}{M^{1/6}}\propto M^{0}\#\left( S28 \right) \end{aligned}$$

If instead muscle force scales with cross sectional area ($F_{musc}\propto M^{2/3}$), the total time for the posture task would scale as:

$$\begin{aligned} t_{ID}={}_{A}t+2{}_{B}t=\frac{c_{1}M^{1}M^{2/3}M^{1/6}}{c_{2}M^{2/3}M^{1/3}M^{1/3}}\propto M^{1/2}.v_{ND}\#\left( S29 \right) \end{aligned}$$

$$\begin{aligned} t_{rel}=\frac{t_{ID}}{t_{char}}\propto\frac{M^{1/2}}{M^{1/6}}\propto M^{1/3}\#\left( S30 \right) \end{aligned}$$

# D. Determining confidence intervals

We used Monte Carlo simulations to determine the confidence intervals of our inertial delay results, based on the confidence intervals of the input parameters. The equations of motion governing the numerical simulations for the swing task are shown in Eqn S31.

$$\begin{aligned} \ddot{\theta}\left( t \right)=\frac{T_{musc}}{MOI}+\frac{M_{limb}gL_{COM}}{MOI}sin \theta\left( t \right)\#\left( S31 \right) \end{aligned}$$

We used the mass, muscle length and moment arm data for the triceps muscle for all non-hoppers reported by Alexander et al. to determine muscle torque $T_{musc}$ [2]. Limb inertial properties including the mass of the limb $M_{limb}$, distance from the shoulder joint to the forelimb COM $L_{COM}$ and forelimb moment of inertia $MOI$ are from Kilbourne and Hoffman [3]. We have reproduced the values for the coefficients and exponents of the power laws for each of these parameters and their confidence intervals in Table 1.

The equations of motion governing the numerical simulations for the posture task are shown in Eqn S32.

$$\begin{aligned} \ddot{\theta}\left( t \right)=\frac{T_{musc}}{M{L_{limb}}^{2}}+\frac{MgL_{limb}}{M{L_{limb}}^{2}}sin \theta\left( t \right)\#\left( S32 \right) \end{aligned}$$

We used the mass, muscle length and moment arm data for the ankle extensor muscles for all non-hoppers reported by Alexander et al. to determine muscle torque $T_{musc}$ [2]. Limb length $L_{limb}$ values are from Kilbourne and Hoffman [3]. We have reproduced the values for the coefficients and exponents of the power laws for each of these parameters and their confidence intervals in Table 2.

## D.1 Creating probability distributions

Kilbourne and Hoffman had reported the 95% confidence intervals for the exponents of the power laws in their paper, but did not do so for the coefficients [3]. Therefore, we processed the raw data provided in the supplementary material to extract this information. We obtained probability distributions for the limb inertial properties using MATLAB’s “fitlm” function, which uses a QR decomposition algorithm to compute a linear regression model to the log transformed raw data.

Since Alexander et al. had 33 specimens in their study to determine muscle properties, we used the reported mean and 95% confidence intervals to create t-distributions (31 d.o.f.) for each muscle property [2].

## D.2 Monte Carlo simulations

We then sampled one value for each of the input parameters from their respective probability distributions and numerically simulated the models to obtain one value for inertial delay. For the limb inertial properties, we used MATLAB’s “random” function to randomly sample from the linear regression model produced by the “fitlm” function, which assumes a normal distribution of data. For the input muscle properties, we used MATLAB’s “trnd” function to randomly sample from the t-distribution.

We ran 10,000 simulations in this way, obtaining a distribution of coefficients and exponents. Our final 95% confidence intervals are 1.96 times the standard deviations of these distributions.


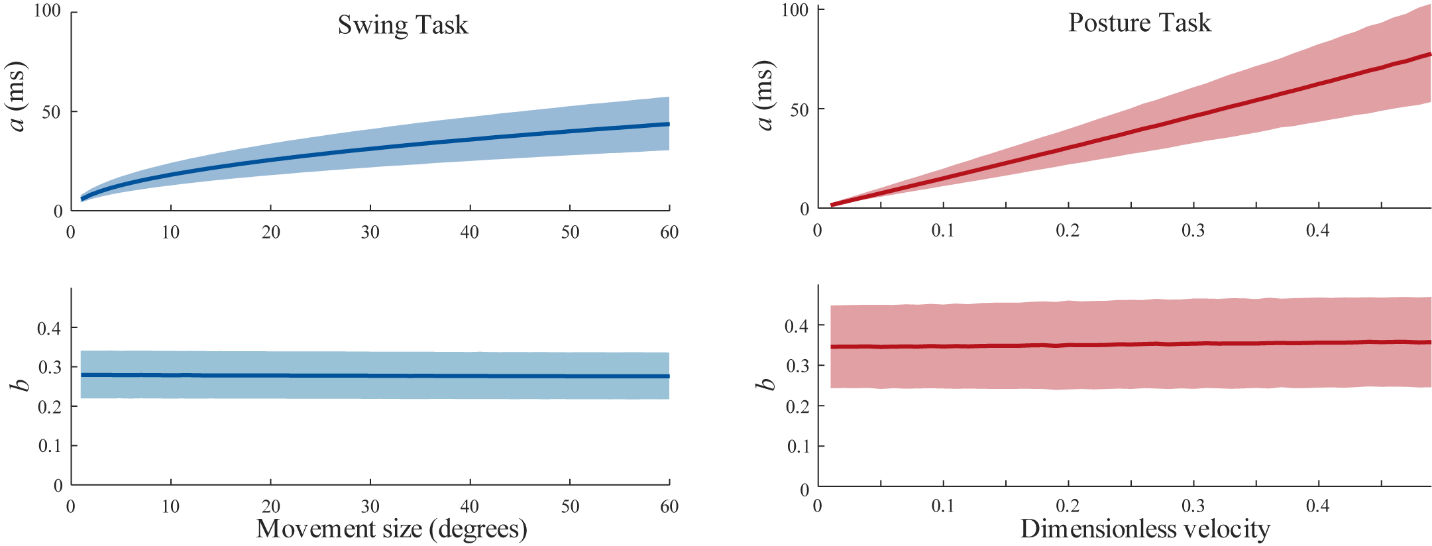


**Fig S1. 95% confidence intervals for the coefficient and exponent of inertial delay.** The shaded region represents the 95% confidence interval for the swing task on the left and the posture task on the right.

# E. Effect of changing muscle torque on inertial delay scaling

Due to sparse information on the scaling of muscles involved in swinging the limb or correcting posture, and widely varying estimates for the isometric force production capacity of mammalian muscle, we were limited in the accuracy of our estimates for muscle torque. Therefore, we tested the sensitivity of our numerical results to the applied muscle torque.

## E.1 Swing Task

We varied the applied torque from zero to four times the original value, for a 30 degree movement in the swing task. The period of an unactuated forelimb scaled as ${281M}^{0.19}$ ms. Inertial delay when using half the original muscle torque value scaled as ${43.3M}^{0.276}$ ms, while at four times the original scaled as ${15.6M}^{0.279}$ ms (Fig S2).


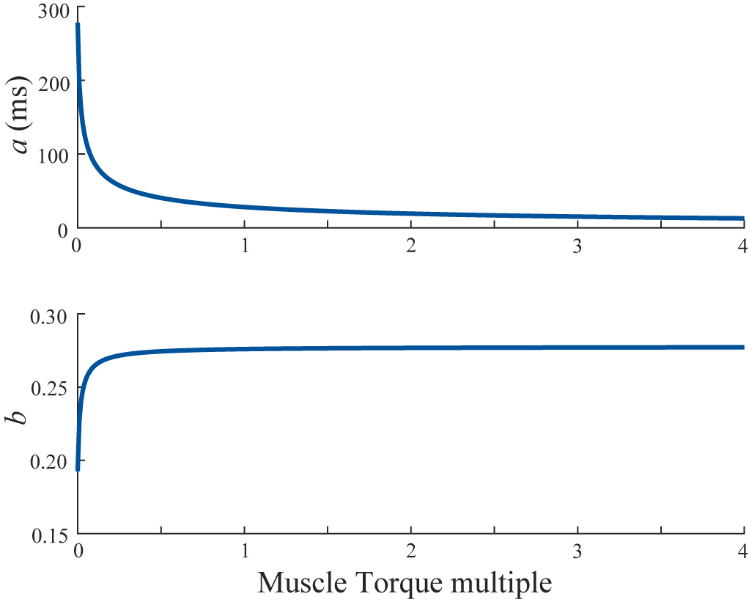


**Fig S2. Effect of muscle torque on the scaling of inertial delay for the swing task.** We varied muscle torque from 0 to 4 times the initial estimate. We show the coefficient and exponent of the power law for a 30 degree movement in the swing task. A value of 0 for muscle torque represents an unactuated pendulum. The exponent levels off at a value of 0.28, while the coefficient decreases with inverse proportionality to the square root of muscle torque.

## E.2 Posture Task

We varied the applied torque from 0.5 to four times the original value, for a perturbation of 0.21 dimensionless velocity for the posture task. If no torque is applied, the inverted pendulum would fall to the floor. Therefore, the lower limit of the range of torques tested was set to 0.5 times the original value. Inertial delay when using half the original muscle torque value scaled as ${64.6M}^{0.363}$ ms, while at four times the original scaled as ${7.6M}^{0.346}$ ms (Fig S3).


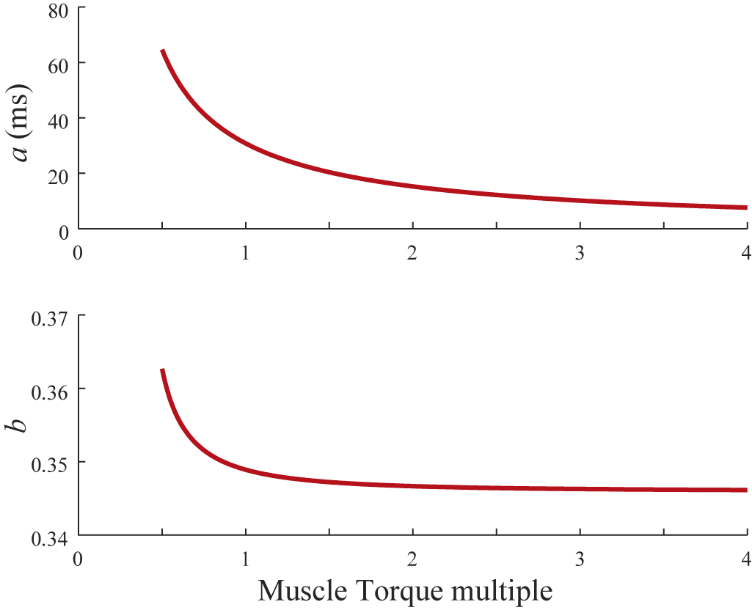


**Fig S3. Effect of muscle torque on the scaling of inertial delay for the posture task.** We varied the muscle torque from 0.5 to 4 times the initial estimate. We show the coefficient and exponent of the power law for a perturbation of 0.21 dimensionless velocity for the posture task. The exponent levels off at a value of 0.346, while the coefficient decreases with inverse proportionality to muscle torque.

# References

1. Hof AL. Scaling gait data to body size. Gait Posture. 1996;3: 222–223. doi:10.1016/0966-6362(95)01057-2

2. Alexander RM, Jayes AS, Maloiy GMO, Wathuta EM. Allometry of the leg muscles of mammals. J Zool. 1981;194: 539–552. doi:10.1111/j.1469-7998.1981.tb04600.x

3. Kilbourne BM, Hoffman LC. Scale effects between body size and limb design in quadrupedal mammals. PLoS One. 2013;8: e78392.
